# The Association of Suicidal Ideation With Family Characteristics and Social Support of the First Batch of Students Returning to a College During the COVID-19 Epidemic Period: A Cross Sectional Study in China

**DOI:** 10.3389/fpsyt.2021.653245

**Published:** 2021-06-28

**Authors:** Yan Chen, Li-jun Zhu, Zheng-mei Fang, Nan Wu, Meng-xue Du, Min-min Jiang, Jing Wang, Ying-shui Yao, Cheng-chao Zhou

**Affiliations:** ^1^Centre for Health Management and Policy Research, School of Public Health, Cheeloo College of Medicine, Shandong University, Jinan, China; ^2^School of Public Health, Wannan Medical College, Wuhu, China; ^3^Anhui College of Traditional Chinese Medicine, Wuhu, China; ^4^National Health Commission (NHC) Key Laboratory of Health Economics and Policy Research, Shandong University, Jinan, China

**Keywords:** COVID-19, college students, suicidal ideation, family characteristics, social support

## Abstract

**Objective:** To investigate the prevalence of suicidal ideation among the first batch of students returning to a college during the COVID-19 epidemic, and to explore the correlation of suicidal ideation with family characteristics and social support.

**Methods:** A cluster sampling survey with a self-designed questionnaire was conducted among the first batch of students returning to a college in Wuhu, China. The Positive and Negative Suicidal ideation (PANSI) and Social Support Scale (SSRS) were used to define students' suicidal ideation and social support, respectively. The influence of family characteristics and social support on the students' suicidal ideation was investigated using multivariate unconditional logistic regression analysis.

**Results:** Two thousand seven hundred valid questionnaires were collected, including 673 males (24.9%) and 2,027 females (75.1%), in this study. A total of 146 students (5.4%) showed suicidal ideation. Male respondents reported higher rates (7.9%) than females (4.6%). Multivariate logistic regression analysis showed that a higher risk level of residence before returning to school and lower objective support were the risk factors for suicidal ideation in males. In contrast, a higher level of maternal education, a poorer relationship with the mother, and lower scores for subjective support and support availability had significant effects on females' suicidal ideation.

**Limitations:** This is a cross-sectional study, and lacks comparison to the time point unaffected by COVID-19. Moreover, it was limited by COVID-19 epidemic prevention and control restrictions, and the differences in returning to school in different regions. Only one college was investigated in this study, and all of the respondents were sophomores, so there may be some limitations in the representativeness of the sample and extrapolation of the results.

**Conclusion:** Family characteristics and social support have had an important influence on suicidal ideation among students returning to school during the COVID-19 epidemic. Some gender differences were identified. Targeted interventions are needed for early prevention and control.

## Introduction

The COVID-19 epidemic swept the world in early 2020, which was listed as a public health emergency of international concern by WHO. The epidemic posing a serious threat to people's lives and safety, and it has created a major mental health challenge (1). Public health measures such as isolation, social distancing, and quarantine were being implemented throughout the world to combat COVID-19. Yet there is evidence suggesting that the specific effects of public health measures implemented to contain disease spread may have a particularly detrimental effect on psychological well-being. It may lead to mental problems such as depression and anxiety, even potentially extending to suicidal ideation and behaviors (2–4) which has aroused the attention of related researchers (5).

Suicide accounts for ~800 000 deaths worldwide annually, which is a serious global public health issue that urgently needs to be addressed (6). In recent years, teenagers have become a high risk group for suicide. Suicide is the most prominent mental health problem among college students, and it has always been the focus of mental health education in colleges and universities (7). Suicide incidences among adolescents and youths during the COVID-19 lockdowns have been reported across the world (8) Remya Lathabhavan (9) reported the first case of student suicide in India due to the COVID-19 education crisis. Mamun (10) also reported the similar case. During the COVID-19 epidemic in China, the government and health department issued various prevention and control policies to curb the spread of the epidemic, and colleges and universities around the country also delayed the opening of school and implemented “online teaching” (11). However, the long-term isolation at home, changes to study and lifestyle, and worry about study and employment have brought great challenges to students' physical and mental health, and various psychological and behavioral problems have become evident (12). At present, international research on the physical and mental health of college students during the epidemic is limited to the period before returning to school. There are no reports on the physical and mental status of students after returning to school, especially with regard to suicide and suicidal ideation.

Now that the Chinese COVID-19 epidemic has gradually shifted to normalized prevention and control, colleges and universities have also begun to resume normal teaching. When students return to “school” from their family, they face changes in their learning and living environment once again, and their physical and mental health are further impacted, which may aggravate the severity and frequency of various psychological and behavioral problems. As the main body responsible for student management, schools should fully understand the importance and complexity of students' mental health problems during the epidemic period, further strengthen and improve mental health education, avoid extreme adverse events, and pay close attention to epidemic prevention and control. These tasks have become the top priority for all colleges that have returned to teaching.

This study selected participants from the first batch of students returning to a college in Wuhu City. A survey was conducted in June 2020 to explore the occurrence of suicidal ideation among the returning students, and the influence of family characteristics and social support on suicidal ideation was analyzed. The aims were to identify the mental health problems of college students after returning to school and propose corresponding countermeasures. This study will provide a theoretical and empirical basis for the positive and effective health promotion measures that can support the physical and mental health of students, reduce the occurrence of suicidal ideation or behavior, help epidemic prevention and control on campus, and promote students' positive physical and mental health.

## Participants and Methods

### Participants

In May 2020, 2,792 students of a college in Wuhu head back to school, and all students were sophomores. For the first batch of returning students, we carried out a cluster sampling survey with a self-designed questionnaire. All of the 2,792 students were investigated taking the class as the unit. Finally, 2,700 valid questionnaires were collected, excluding 92 questionnaires with incomplete information (Effective response rate = 96.7%).

### Measures

#### General Demographic Information and Family Characteristics

Information was obtained on general demographic variables: gender, age, grade, major, native place, nationality.

Family characteristics, such as home location, family type, only-child or not, parents' occupations, parents' educational levels, family relationship, and parental expectations for students, were collected. Three additional questions were set in the questionnaire: “whether the parents were front-line workers;” “whether the family had suffered economic losses during the epidemic period,” and “the risk level of the residence before returning to school.”

#### Positive and Negative Suicidal Ideation Questionnaire

In this study, students' suicidal ideation was measured by the Positive and Negative Suicidal ideation (PANSI) scale revised by Ying-Nan Liang from Osman (13, 14). PANSI is a validated and widely used measure of suicidal ideation in college students. Respondents report the frequency of symptoms experienced within the last 2 weeks. The scale consists of two dimensions: Positive suicidal ideation (six items, PI) and Negative suicidal ideation (eight items, NI). Each item was rated on a five-point scale (“1—never had” to “5—always”). The average scores of PI and NI were both ranged from 1 to 5, and suicidal ideation was determined with NI score threshold value ≥1.625 and PI score threshold value ≤3.333 as the boundary (13). The scale has good reliability and validity (retest reliability = 0.865).

#### Social Support Scale (SSRS)

The Social Support Scale was compiled by Xiao Shuiyuan (15, 16). It has 10 items, which are divided into three dimensions: Objective support, Subjective support, and Utilization of social support. The sum of the three scores equals the total score for Social Support. The higher the total score, the greater the social support available. The scale has good reliability and validity and has been widely used (retest reliability = 0.92).

### Quality Control

A preliminary investigation was conducted before the survey and the questionnaire was modified and improved according to the findings. A formal investigation was carried out by investigators with unified training. Before the investigation began, all respondents signed the informed consent form and, at the same time, the investigators gave the corresponding guidance. The questionnaires were collected directly from participants after completion and checked in a timely manner.

### Statistical Analysis

The data were inputted into a database established using Epidata 3.0 software, and analyzed using IBM SPSS 26.0. Measurement data were described by mean ± standard deviation, and the comparison between groups was conducted by *t* test; the count data were described by rate or composition ratio, and the comparison between groups was conducted by the chi-square test. The rank sum test was used to compare data with a skewed distribution or hierarchical data between different groups. Multivariate logistic regression analysis was used to explore the correlation between suicidal ideation and family characteristics. Bilateral testing was used, with the test level α = 0.05.

## Results

### Comparison of Family Characteristics, Social Support, and Suicidal Ideation Between Students of Different Genders

In this study, 2,700 valid questionnaires were collected, including 673 male (24.9%) and 2,027 female students (75.1%), with an average age of 20.5 ± 0.96 years old. According to the PANSI, a total of 146 students (5.4%) showed suicidal ideation. Male respondents reported higher rates (53, 7.9%) than females (93, 4.6%). Family characteristics had significant effects on suicidal ideation which varied between the genders, including native place, home location, only child or not, father's and mother's educational level, father's and mother's career, parental expectation, and whether the family had suffered economic losses during the epidemic period (*P* < 0.05). In terms of social support, availability of support was higher for females than for males (*P* < 0.05), the detailed results are shown in Table 1.

**Table 1:** Comparison of family characteristics, social support and suicidal ideation between male and female students (*n* = 2,700).

**Variables**		**Males*n* = 673 (%)**	**Females*n* = 2,027 (%)**	***Z/*χ^2^**	***P***	**Variables**		**Males*n* = 673 (%)**	**Females *n* = 2,027 (%)**	***Z/*χ^2^*/t***	***P***
Nation	Han	650 (96.6)	1,955 (96.4)	0.027	0.870	Parents' relationship	Very bad	6 (0.9)	13 (0.6)	−0.359	0.720
	Others	23 (3.4)	72 (3.6)				Bad	12 (1.8)	17 (0.8)		
Native place	Anhui	497 (73.8)	1,694 (83.6)	31.225	<0.001		Gerneral	73 (10.8)	238 (11.7)		
	Others	176 (26.2)	333 (16.4)				Good	192 (28.5)	610 (30.1)		
Home location	Rural	446 (66.3)	1,505 (74.2)	16.040	<0.001		Very good	390 (57.9)	1,149 (56.7)		
	City	227 (33.7)	522 (25.8)			Relationship with father	Very bad	3 (0.4)	8 (0.4)	−0.604	0.546
only-child or not	Yes	291 (43.2)	385 (19.0)	158.250	<0.001		Bad	8 (1.2)	15 (0.7)		
	No	382 (56.8)	1,642 (81.0)				Gerneral	83 (12.3)	236 (11.6)		
Father's educational level	≤ Primary school	145 (21.5)	528 (26.0)	41.575	<0.001		Good	191 (28.4)	645 (31.8)		
	Junior high school	298 (44.3)	1,027 (50.7)				Very good	388 (57.7)	1,123 (55.4)		
	High school	130 (19.3)	316 (15.6)			Relationship with mother	Very bad	2 (0.3)	3 (0.1)	−0.180	0.857
	Higher vocational colleges	65 (9.7)	112 (5.5)				Bad	3 (0.4)	6 (0.3)		
	≥Bachelor	35 (5.2)	44 (2.2)				Gerneral	52 (7.7)	138 (6.8)		
Mother's educational level	≤ Primary school	253 (37.6)	958 (47.3)	34.489	<0.001		Good	179 (26.6)	565 (27.9)		
	Junior high school	259 (38.5)	737 (36.4)				Very good	437 (64.9)	1,315 (64.9)		
	High school	96 (14.3)	228 (11.2)			Parents' expectations	Very high	83 (12.3)	146 (7.2)	−4.139	<0.001
	Higher vocational colleges	43 (6.4)	80 (3.9)				High	381 (56.6)	1,116 (55.1)		
	≥Bachelor	22 (3.3)	24 (1.2)				Gerneral	201 (29.9)	733 (36.2)		
Father's career	Farmers	164 (24.4)	606 (29.9)	44.128	<0.001		Low	4 (0.6)	22 (1.1)		
	Workers	198 (29.4)	640 (31.6)				Very low	4 (0.6)	10 (0.5)		
	Public functionary	82 (12.2)	103 (5.1)			Economic loss of family	Yes	377 (56.0)	1,343 (66.3)	22.872	<0.001
	Hobo	26 (3.9)	64 (3.2)				No	295 (43.8)	683 (33.7)		
	Others	203 (30.2)	614 (30.3)			Risk level of residence before returning to school	Higher	4 (0.6)	9 (0.4)	−0.485	0.627
Mother's career	Farmers	173(25.7)	664(32.8)	30.119	<0.001		High	8(1.2)	13(0.6)		
	Workers	127 (18.9)	306 (15.1)				Gerneral	21 (3.1)	36 (1.8)		
	Public functionary	54 (8.0)	83 (4.1)				Low	51 (7.6)	216 (10.7)		
	Hobo	116 (17.2)	399 (19.7)				Lower	589 (87.5)	1,753 (86.5)		
	Others	203 (30.2)	575 (28.4)			Suicidal ideation	No	620 (92.1)	1,934 (95.4)	10.673	0.001
Family types	Nuclear family	488 (72.5)	1,406 (69.4)	3.004	0.391		Yes	53 (7.9)	93 (4.6)		
	Single parent family	38 (5.6)	126 (6.2)			social support	Subjective support	20.88 ± 4.06	20.64 ± 3.50	1.380	0.168
	Three generations	133 (19.8)	436 (21.5)				Objective support	9.78 ± 2.56	9.69 ± 2.26	0.830	0.407
	Remarriage and others	14 (2.1)	59 (2.9)				Support availability	8.01 ± 1.97	8.43 ± 1.73	4.904	<0.001
Front-line anti-epidemic workers	Father	7 (1.0)	9 (0.4)	4.037	0.257		Total social support	38.67 ± 6.61	38.75 ± 5.57	0.294	0.768
	Mother	2 (0.3)	4 (0.2)								
	Both father and mother	4 (0.6)	6 (0.3)								
	None	660 (98.1)	2,008 (99.1)								

### Univariate Logistic Regression Analysis

The results of the univariate analysis showed that suicidal ideation in students was influenced by family characteristics and social support. Gender shared some common factors, but gender differences were evident. Relationship with mother, risk level of residence before returning to school, subjective support, objective support, support availability, and total social support were the common influencing factors for both male and female students (*P* < 0.05). In addition, mother's education level, parents' relationship, relationship with father, and parental expectations were independent factors influencing suicidal ideation in females (*P* < 0.05). See Table 2 and Supplementary Table 2.

**Table 2:** Univariate logistic regression analysis of suicidal ideation.

**Varibles**	***B***	***S.E*.**	***Waldχ^2^***	***P***	***OR***	***95%CI***
**Males**						
Relationship with mother	−0.389	0.181	4.628	0.031	0.677	0.475–0.966
Risk level of residence before returning to school	−0.672	0.155	5.080	0.024	0.714	0.532–0.957
Subjective support	−0.141	0.035	16.054	<0.001	0.868	0.810–0.930
Objective support	−0.266	0.060	19.563	<0.001	0.766	0.681–0.862
Support availability	−0.153	0.076	4.012	0.045	0.858	0.739–0.997
Total social support	−0.104	0.022	22.248	<0.001	0.901	0.863–0.941
**Females**						
Mother's education level	0.228	0.107	4.507	0.034	1.256	1.018–1.550
Parents' relationship	−0.576	0.110	27.293	<0.001	0.562	0.453–0.698
Relationship with father	−0.638	0.116	30.124	<0.001	0.528	0.421–0.663
Relationship with mother	−0.906	0.129	49.722	<0.001	0.404	0.314–0.520
Parental expectations	0.309	0.161	3.703	0.054	1.362	0.994–1.866
Risk level of residence before returning to school	−0.338	0.150	5.080	0.024	0.714	0.532–0.957
Subjective support	−0.199	0.031	42.383	<0.001	0.820	0.772–0.870
Objective support	−0.223	0.050	19.753	<0.001	0.800	0.726–0.883
Support availability	−0.433	0.071	37.657	<0.001	0.649	0.565–0.745
Total social support	−0.147	0.019	57.721	<0.001	0.864	0.831–0.897

### Multivariate Logistic Regression Analysis

Taking the suicidal ideation of male and female students as the dependent variable, and the significant variables in the univariate analysis related to family characteristics and social support as the independent variables, the multivariate logistic regression analysis was conducted using the variable assignment shown in Supplementary Table 1. The results showed that a higher risk level of the residence before returning to school and lower objective support were the risk factors for suicidal ideation in males. In contrast, a higher level of maternal education, a poorer relationship with the mother, and lower scores for subjective support and support availability had significant effects on suicidal ideation in females (Table 3).

**Table 3:** Multivariate logistic regression analysis of suicidal ideation.

**Varibles**	***B***	***S.E*.**	***Waldχ^2^***	***P***	***OR***	***95%CI***
**Males**						
Risk level of residence before returning to school	−0.559	0.164	11.684	0.001	0.572	0.415–0.788
Objective support	−0.191	0.067	8.032	0.005	0.826	0.724–0.943
Constant	3.878	1.201	10.429	0.001	48.336	
**Females**						
Mother's education level	0.246	0.111	4.886	0.027	1.279	1.028–1.591
Relationship with mother	−0.682	0.200	11.599	0.001	0.506	0.341–0.749
Subjective support	−0.085	0.036	5.722	0.017	0.918	0.857–0.985
Support availability	−0.266	0.076	12.145	<0.001	0.767	0.660–0.890
Constant	5.480	1.107	24.512	<0.001	239.773	

## Discussion

Among the 2,700 participants from the Higher vocational college in Wuhu, 146 (5.4%) students showed suicidal ideation. This finding is not in line with the suicide rates observed during previous pandemics. Kaparounaki (17) carried out a study of the mental health status of 1,000 college students in Greece found that, 63.3% increase in total suicidal thoughts in a population from the first 1,000 university students during the COVID-19 quarantine period. In comparison, Wang reported suicidal ideation among 18.04% of US college students during the COVID-19 pandemic (18). The reason may be that the incidence of suicidal ideation is not consistent due to the different methods used to measure suicidal ideation in different studies. In addition, the different research objects, school types, major and social environment will also affect the occurrence of suicidal ideation of college students. Shen Ke (19) reported the detection rate of combined suicidal ideation among medical students in mainland China from 2007 to 2020 (11.73%) through a meta-analysis. The results of this study are lower than the national level of medical students, and it is at a low level in general, which may be related to the adequate prevention and control measures and timely psychological intervention of the school.

The early identification of influencing factors which affect students' suicidal ideation and targeted intervention are the main measures used to prevent students from committing suicide (20, 21). The results of Chinese and international research on suicidal ideation in male and female students are inconsistent. Some studies found that females have higher rates of suicidal ideation than males (22, 23), while other research indicates that males have a higher suicidal ideation rate (24). Also, some studies argue there are no gender differences (25). The results of this study showed that male students have a higher suicidal ideation rate than female students. The reason may be related to recent improvements in women's societal status which has led to a healthier self-identity, and a lower suicide rate as a consequence. This means that gender differences in suicidal ideation may have gradually weakened in recent years (26).

The family environment plays an important role in the personal development and growth of college students. Previous studies (27–29); found that family type, family relationship, family economics, parental attention, parents' occupations, family location, and whether an only child or not, may have an important impact on suicidal ideation. Our previous research (30) indicated that there is a significant correlation between family relationship and economic level and college students' suicide behaviors. This study found that a worse relationship with the mother and a higher maternal education level were risk factors for females, which may be because females are more sensitive to family relationships than males. Also, the relationship between the mother and student has a more obvious influence, as primary caregiver, on a student's suicidal ideation, especially for females. Students may come under more pressure from mothers who have higher educational levels. The results of this study did not show differences associated with family type, parents' occupations, education level, family location, and whether an only child or not. However, as common factors affecting suicidal ideation and suicidal behavior of students, these should not be ignored, especially for students from vulnerable groups such as single-parent families.

Among the three family factors specifically designed for the COVID-19 epidemic, the risk level of the residence before returning to school was significantly associated with suicidal ideation in males, suggesting that schools need to pay special attention to students from high-risk areas. However, there were no significant differences in whether the parents were front-line, anti-epidemic workers and whether the family had suffered economic losses during the epidemic period. The number of students who had frontline anti-epidemic parents was small (32 respondents), and the possibility of low test efficiency cannot be ruled out. Moreover, the impact of family economic losses during the epidemic on the rate of students' suicidal ideation may not have an immediate or short-term influence, and this needs to be verified in the longer term.

The results of this study also showed that social support is an important influencing factor for suicidal ideation in both males and females, which is consistent with the results of other similar studies in China and internationally (31–34). An interesting finding is that the influence of social support on suicidal ideation were inconsistent between genders. For male students, objective support was the statistically significant influencing factor. In contrast, subjective support and support availability were the significant factors for females. This suggests that we should establish a targeted support system for students of different genders. Good social support is an important factor in reducing the suicide risk of students. When encountering setbacks and difficulties, positive support from the family, school, and society in general will lead to more positive emotional responses and motivation, and this can help a student's capacity to find solutions without support, students may experience more negative emotions, which will lead to more self-denial and self-isolation, and even to suicidal ideation.

To summarize, it will be beneficial if schools cooperate with parents to change students' living and learning environments, further strengthen parent–child communication, and create a good family atmosphere for students. At the same time, it is necessary to identify high-risk students as early as possible, carry out targeted health education, and provide more social support for students from high-risk areas to support mental health, and prevent suicidal behavior.

## Limitations

This was a cross-sectional study and it lacks a comparison to the time point unaffected by COVID-19. Moreover, it was limited to the specific circumstances of the COVID-19 prevention and control measures, and by the differences in the situation of returning to school in different regions. Only one college was investigated in this study and all respondents were sophomores, so there may be some limitations in the representativeness of the sample and extrapolation of the results.

## Data Availability Statement

The raw data supporting the conclusions of this article will be made available by the authors, without undue reservation.

## Ethics Statement

The studies involving human participants were reviewed and approved by School of Public Health of Wannan Medical College. The patients/participants provided their written informed consent to participate in this study.

## Author Contributions

YC and C-cZ: conception and design. Y-sY: administrative support and provision of research objects. L-jZ, Z-mF, NW, M-xD, M-mJ, and JW: collection and assembly of data. YC and Y-sY: data analysis and interpretation. YC: the first draft of the manuscript writing. C-cZ: results evaluation and manuscript revision. All authors: final approval of manuscript.

## Conflict of Interest

The authors declare that the research was conducted in the absence of any commercial or financial relationships that could be construed as a potential conflict of interest.
