# Supplementary material for: The Association of Suicidal Ideation With Family Characteristics and Social Support of the First Batch of Students Returning to a College During the COVID-19 Epidemic Period: A Cross Sectional Study in China
Source: Front Psychiatry. 2021 Jun 28;12:653245. doi: 10.3389/fpsyt.2021.653245 (PMC8273509; doi:10.3389/fpsyt.2021.653245)
Supplement: Supplementary file 1 [file Data_Sheet_1.pdf]

Supplementary Table 1 Description of independent variable assignment

Variable	Assignment	Model inclusion mode
Gender	0=Males，1=Females	Original data
Nationality	0= The han nationality，1=Others	Original data
Nativeplace	0=Anhui，1=Others	Original data
Home location	0=Rural，1=City	Original data
Family types	1=Nuclear family，2= Single parent families，3= Three generations，4= Remarriage and others	Dummy variable（reference=1）
Only-child or not	0=No，1=Yes	Original data
Father's education level	1=Primary schools and below，2=Junior high school，3=high school，4= Higher vocational colleges，5= Bachelor and above	Original data
Mother's education level	1=Primary schools and below，2=Junior high school，3=high school，4= Higher vocational colleges，5= Bachelor and above	Original data
Father's career	1=Farmers，2=Workers，3= Staff of public institutions，4=Hobo，5=others	Dummy variable（reference =1）
Mother's career	1=Farmers，2=Workers，3= Staff of public institutions，4=Hobo，5=others	Dummy variable（reference =1）
Front-line anti-epidemic workers	1=father，2=mother，3=both father and mother，4=none	Dummy variable（reference =4）
Parents' relationship	1=very bad，2=bad，3=gerneral，4=good，5=very good	Original data
Relationship with father	1=very bad，2=bad，3=gerneral，4=good，5=very good	Original data
Relationship with mother	1=very bad，2=bad，3=gerneral，4=good，5=very good	Original data
Parental expectations	1=very high, 2=high，3= gerneral，4=low，5=very low	Original data
Economic losses of family during the epidemic	0=No，1=Yes	Original data
Risk level of residence before returning to school	1=higher risk，2=high risk，3=medium risk，4=low risk，5=lower risk	Original data
Subjective support	——	Original data
Objective support	——	Original data
Support availability	——	Original data
Total social support	——	Original data

Supplementary Table 2 Univariate logistic regression analysis of suicidal ideation

Varibles		*B*	*S.E.*	Waldχ^2	*P*	*OR*	95%CI
**Males**							
Nationality	Others	-0.112	0.754	0.022	0.882	0.894	0.204~3.921
Nativeplace	Others	-0.738	0.394	3.500	0.061	0.478	0.221~1.036
Home location	City	0.102	0.299	0.116	0.734	1.107	0.616~1.988
Family type	Nuclear family			0.029	0.999		
	Single parent family	0.176	1.051	0.028	0.867	1.192	0.152~9.346
	Three generations	-18638.	6520.161	0.000	0.998	0.000	0.00
	Remarriage and others	0.159	1.084	0.021	0.884	1.172	0.140~9.819
Only-child or not	Yes	-0.336	0.298	1.271	0.260	0.715	0.399~1.281
Father's education level		0.146	0.127	1.327	0.249	1.158	0.902~1.485
Mother's education level		0.232	0.126	3.391	0.066	1.261	0.985~1.615
Father's career	Farmers			2.677	0.613		
	Workers	0.143	0.415	0.119	0.730	1.154	0.512~2.602
	Staff of public institutions	0.379	0.379	1.005	0.316	1.462	0.696~3.069
	Hobo	0.589	0.455	1.675	0.196	1.802	0.739~4.395
	Others	-0.537	1.059	0.257	0.612	0.585	0.073~4.662
Mother's career	Farmers			3.656	0.455		
	Workers	0.505	0.411	1.508	0.219	1.657	0.740~3.710
	Staff of public institutions	0.688	0.426	2.606	0.106	1.990	0.863~4.591
	Hobo	0.780	0.533	2.146	0.143	2.182	0.768~6.196
	Others	0.257	0.480	0.286	0.592	1.293	0.505~3.312
Front-line anti-epidemic workers	father			3.390	0.335		
	mother	1.564	0.849	3.390	0.066	4.776	0.904~25.235
	both	-18.723	28420.722	0.000	0.999	0.000	0.000
	none	-18.723	20096.485	0.000	0.999	0.000	0.000
Parents' relationship		-0.210	0.158	1.769	0.183	0.810	0.594~1.105
Relationship with father		-0.299	0.164	3.315	0.069	0.742	0.538~1.023
Relationship with mother		-0.389	0.181	4.628	0.031	0.677	0.475~0.966
Parental expectations		0.269	0.207	1.683	0.195	1.308	0.872~1.962
Economic losses of family during the epidemic	Yes	0.191	0.292	0.426	0.514	1.210	0.682~2.146
Risk level of residence before returning to school		-0.672	0.155	5.080	0.024	0.714	0.532~0.957
Subjective support		-0.141	0.035	16.054	0.000	0.868	0.810~0.930
Objective support		-0.266	0.060	19.563	0.000	0.766	0.681~0.862
Support availability		-0.153	0.076	4.012	0.045	0.858	0.739~0.997
Total social support		-0.104	0.022	22.248	0.000	0.901	0.863~0.941

**Females**							
Nationality	Others	-0.210	0.526	0.159	0.690	0.811	0.289~2.722
Nativeplace	Others	-0.108	0.296	0.134	0.714	0.897	0.502~1.604
Home location	City	0.118	0.237	0.248	0.619	1.125	0.707~1.790
Family type	Nuclear family			6.359	0.095		
	Single parent family	-0.022	0.605	0.001	0.971	0.978	0.299~3.202
	Three generations	0.362	0.686	0.278	0.598	1.436	0.374~5.511
	Remarriage and others	-0.727	0.667	1.191	0.275	0.483	0.131~1.785
Only-child or not	Yes	0.416	0.244	2.903	0.088	1.516	0.939~2.445
Father's education level		0.097	0.112	0.743	0.389	1.102	0.884~1.373
Mother's education level		0.228	0.107	4.507	0.034	1.256	1.018~1.550
Father's career	Farmers			0.465	0.977		
	Workers	0.053	0.281	0.036	0.850	1.055	0.608~1.829
	Staff of public institutions	0.174	0.270	0.415	0.519	1.190	0.701~2.022
	Hobo	0.143	0.500	0.082	0.775	1.154	0.433~3.077
	Others	0.106	0.624	0.029	0.865	1.112	0.327~3.782
Mother's career	Farmers			0.739	0.946		
	Workers	0.117	0.278	0.178	0.673	1.124	0.652~1.939
	Staff of public institutions	0.018	0.352	0.003	0.958	1.019	0.511~2.030
	Hobo	0.151	0.553	0.074	0.786	1.162	0.393~3.438
	Others	0.243	0.306	0.631	0.427	1.275	0.700~2.324
Front-line anti-epidemic workers	father			1.680	0.641		
	mother	-18.167	13397.657	0.000	0.999	0.000	0.000
	both	-18.167	20096.485	0.000	0.999	0.000	0.000
	none	1.427	1.101	1.680	0.195	4.165	0.482~36.016
Parents' relationship		-0.576	0.110	27.293	0.000	0.562	0.453~0.698
Relationship with father		-0.638	0.116	30.124	0.000	0.528	0.421~0.663
Relationship with mother		-0.906	0.129	49.722	0.000	0.404	0.314~0.520
Parental expectations		0.309	0.161	3.703	0.054	1.362	0.994~1.866
Economic losses of family during the epidemic	Yes	0.121	0.229	0.279	0.598	1.128	0.720~1.768
Risk level of residence before returning to school		-0.338	0.150	5.080	0.024	0.714	0.532~0.957
Subjective support		-0.199	0.031	42.383	0.000	0.820	0.772~0.870
Objective support		-0.223	0.050	19.753	0.000	0.800	0.726~0.883
Support availability		-0.433	0.071	37.657	0.000	0.649	0.565~0.745
Total social support		-0.147	0.019	57.721	0.000	0.864	0.831~0.897
